# Age-Related Changes of Gene Expression Profiles in *Drosophila*

**DOI:** 10.3390/genes12121982

**Published:** 2021-12-14

**Authors:** Guillaume Bordet, Niraj Lodhi, Andrew Kossenkov, Alexei Tulin

**Affiliations:** 1 Department of Biomedical Sciences, School of Medicine and Health Sciences, University of North Dakota, 501 North Columbia Road, Stop 9061, Grand Forks, ND 58202, USA; Guillaume.Bordet@und.edu; 2 Basic Science Department, Fox Chase Cancer Center, Philadelphia, PA 19111, USA; lodhiniraj@gmail.com; 3 Gene Expression & Regulation Program, The Wistar Institute Cancer Center, Philadelphia, PA 19104, USA; akossenkov@Wistar.org

**Keywords:** aging processes, microarray, *Drosophila*, muscle structure, cytochrome

## Abstract

An individual’s gene expression profile changes throughout their life. This change in gene expression is shaped by differences in physiological needs and functions between the younger and older organism. Despite intensive studies, the aging process is not fully understood, and several genes involved in this process may remain to be identified. Here we report a transcriptomic analysis of *Drosophila melanogaster* using microarrays. We compared the expression profiles of two-day-old female adult flies with those of 45-day-old flies. We identified 1184 genes with pronounced differences in expression level between young and old age groups. Most genes involved in muscle development/maintenance that display different levels of expression with age were downregulated in older flies. Many of these genes contributed to sarcomere formation and function. Several of these genes were functionally related to direct and indirect flight muscles; some of them were exclusively expressed in these muscles. Conversely, several genes involved in apoptosis processes were upregulated in aging flies. In addition, several genes involved in resistance to toxic chemicals were upregulated in aging flies, which is consistent with a global upregulation of the defense response system in aging flies. Finally, we randomly selected 12 genes among 232 genes with unknown function and generated transgenic flies expressing recombinant proteins fused with GFP protein to determine their subcellular expression. We also found that the knockdown of some of those 12 genes can affect the lifespan of flies.

## 1. Introduction

Aging of an organism progresses with cascades of molecular changes, some of which are universal across all eukaryotes, while others are specific to taxonomic units [1,2,3,4,5,6,7,8,9]. In a living cell, these molecular changes regulate the transcriptional activation of age-related genes, the capacity for DNA repair, and programmed cell death, thereby mediating rates of cellular proliferation and differentiation. Coupled with environmental stress and damage accumulation, such changes lead to an exponential increase in frailty and morbidity over time, ultimately determining the lifespan of an organism.

With an increasing proportion of older individuals in industrialized human populations, understanding the underlying mechanisms of aging- and senescence-associated diseases has gained tremendous clinical importance. Aging-related morbidity involves an increase in the risk for certain cancers [1,2], heart disease [3], macular degeneration [4], Alzheimer’s disease [5], osteoporosis [6], and a plethora of other conditions.

*D. melanogaster* is a suitable model organism for studying aging mechanisms because it has a short lifespan (less than 3 months) [10], and individuals are easy to cultivate. To better understand the mechanisms involved in aging processes, we compared the expression profile of two-day-old adult female flies with the expression profile of 45-day-old flies. For this study, we followed a whole organism approach to detect large-scale organismal changes in the expression profiles of all genes, rather than tissue-specific changes. We identified 1184 genes differentially expressed between young and aging flies. Our statistical analysis establishes which functional gene groups were overrepresented among these genes and upregulated at specific ages, linking their variable expression to the progression of aging. In total, 232 of these genes had no known function. We have randomly selected 12 of these genes and analyzed the cellular localization of their products and the consequences of transgenic knockdowns on fly longevity.

## 2. Results

### 2.1. Comparison of the Expression Profile between Young and Old Age Groups

To compare the expression profiles of young and old age fruit flies, we collected y(1), w67c23(2) female flies at day three and day 45 post-pupation (hereinafter termed as young and old age groups, respectively). Flies were mated with males for two days after pupation and then cultured at 20 °C for the duration of the experiment. We selected 45-day-old flies as the old age group because fly survivorship remains high until this time point and decreases sharply after it [10]. This survivorship decrease is linked with the accumulation of necrotic and apoptotic tissues, which might cause changes in the transcriptomic profile, masking partly the changes that occur due to developmental changes. The expression profile was determined using a microarray chip and was realized in three biological replicates to control for random fluctuations. The fluorescence intensity of each probe is presented in our raw data as a log_2_ value (Supplementary Table S1). Differentially expressed genes (DEGs) have been identified based on a the following criteria: (1) fold difference higher than two, (2) two-tailed *t*-test based on change of expression between young and old age groups relative to the standard deviation of all measurements with a resulting *p*-value lower than 0.05, and (3) false discovery rate (FDR) lower than 15% (see Section 4).

**Figure 1:**
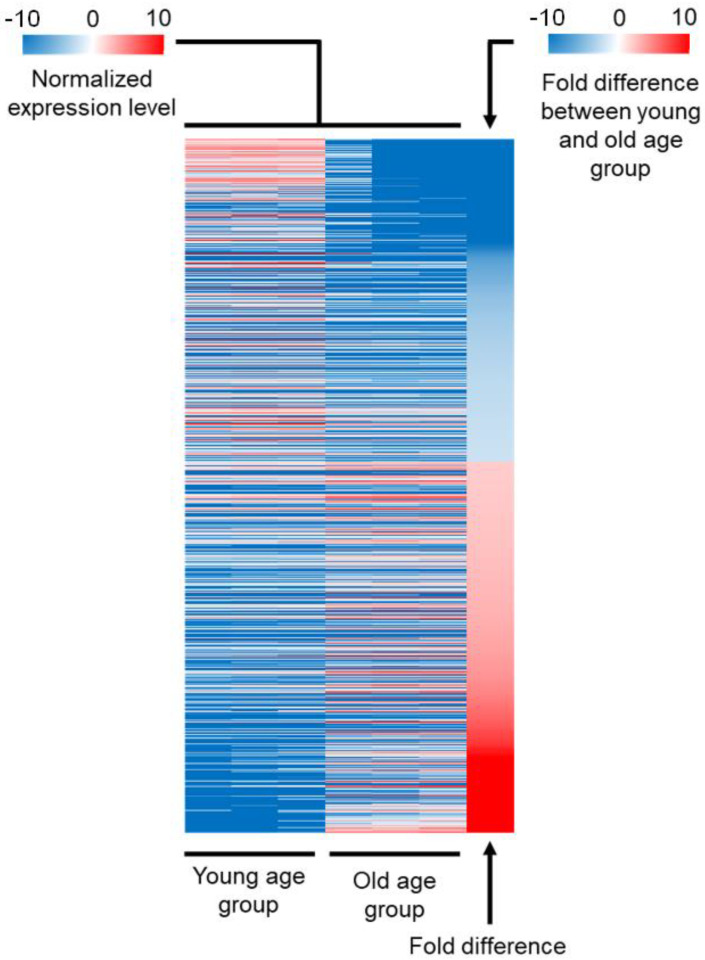
Overview of the transcriptomic analysis. Heatmap representing the expression level of DEGs between old and young age groups that followed our three criteria: (1) fold difference higher than 2, (2) *p*-value of *t*-test based on three biological replicates lower than 0.05, and (3) false discovery rate (FDR) lower than 15%. The three biological replicates are shown separately. The expression level of each gene is normalized by subtracting the average level of expression for all genes. A negative value corresponds to a gene that is downregulated in old age flies (downregulated DEGs) (blue), while a positive value corresponds to a gene that is upregulated in old age flies (upregulated DEGs) (red). The last column represents the fold difference between old and young age groups. The value is negative when the expression is higher in the young age group (blue), and the value is positive when the expression is higher in the old age group (red).

Our analysis identified 1225 probes that presented differential fluorescence intensity between young and old age groups. We identified 1147 genes targeted by a unique probe and 37 genes targeted by more than one probe, leading to 1184 genes differentially expressed between young and old age groups. In total, 550 of these genes were downregulated in old age flies (hereinafter termed as “downregulated DEGs”), whereas the remaining 634 were upregulated (hereinafter termed as “upregulated DEGs”). Probes targeting the same gene were all upregulated or downregulated in old age flies. The three biological replicates produced similar results for each gene, suggesting that the expression level is stable in an age-constrained group (Figure 1). The absolute range of expression level was extremely broad between young and old age groups, from negative 559-fold for the cuticle protein coding gene Acp65Aa to 591-fold for the NPC2-like gene Npc2e. However, the fold difference of 88% of these 1184 genes ranged between −25 and 75 only (Supplementary Figure S1). To determine which cellular functions were more affected by aging than others, we next carried out a functional analysis of DEGs.

### 2.2. Cellular Functions of DEGs

**Figure 2:**
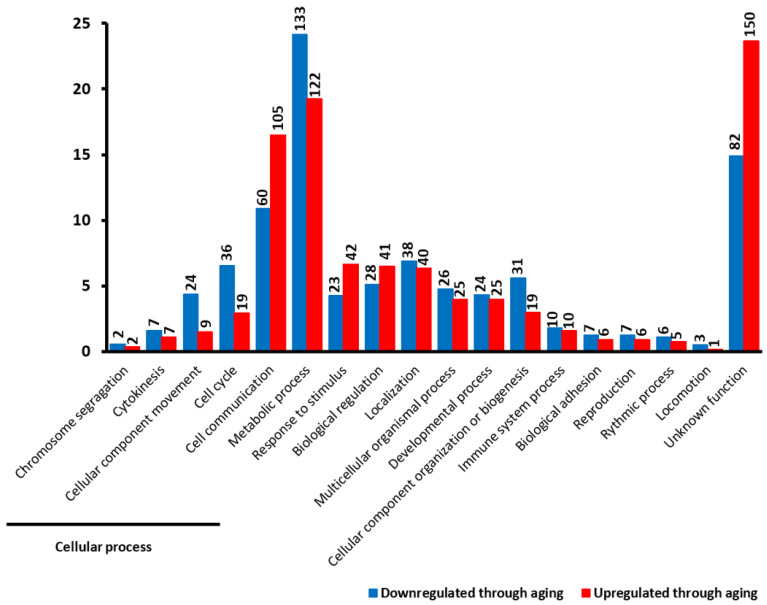
Overview of main cellular functions of 1184 differentially expressed genes between young and old age groups. The first five processes are subcategories of the “cellular process”. The height of each bar corresponds to the percentage of genes belonging to each specific process. Genes downregulated in the old age group are represented in blue, whereas genes upregulated in the old age group are represented in red. The black number at the top of each bar corresponds to the number of DEGs belonging to each specific process.

To identify the functions of DEGs, we tested the list with 1184 genes using the PANTHER classification system [11] (see Section 4). Among the 1184 DEGs, 952 possess a known cellular function, whereas the function of the other 232 genes is unknown. These genes with unknown function correspond to 82 out of 550 (14.9%) of the downregulated DEGs and 150 out of 634 (23.7%) of the upregulated DEGs (Figure 2). The 952 genes with known functions are involved in a range of cellular functions. We identified five categories represented in different proportions among upregulated and downregulated DEGs. The “cellular process” category is the most represented in that 22.4% of the upregulated DEGs and 25% of the downregulated DEGs fall into this category. Among upregulated DEGs involved in the “cellular process”, 73.8% of them play a role in “cell-cell communication”, compared to 45.5% for downregulated DEGs. Other upregulated DEGs that play a role in the“cellular process” are involved in the cell cycle (13.1%) and “cellular component movement” (6.6%), compared to downregulated DEGs (27.3% and 18.2%, respectively).

We identified other categories of cellular function represented in different proportions between upregulated and downregulated DEGs, including “response to stimulus” (6.6% and 4.3% for upregulated and downregulated DEGs, respectively), which are genes that respond to stress or genes involved in cellular and immune defense, and “biological regulation” (6.5% and 5.1%), including genes that regulate catalytic activity or play a role in the regulation of nucleobase-containing compound metabolism.

In addition, we found several categories represented in similar proportions among upregulated and downregulated DEGs, including “localization” (6.3% and 6.9 for upregulated and downregulated DEGS, respectively), “multicellular organismal process” (4% and 4.8%), “developmental process” (4% and 4.3%), “immune system process” (1.6% and 1.8%), and other categories (Figure 2). Taken together, these results suggest that some cellular functions are differentially regulated between young and old age groups. The genes important for the processes involved in defense response and biological regulation seem to be upregulated in the old age group, whereas genes important for cellular component organization or biogenesis are downregulated. However, this method does not tell us if a particular category is significantly overrepresented among the DEGs.

### 2.3. Distribution of DEG Types between Young and Old Age Groups

**Figure 3:**
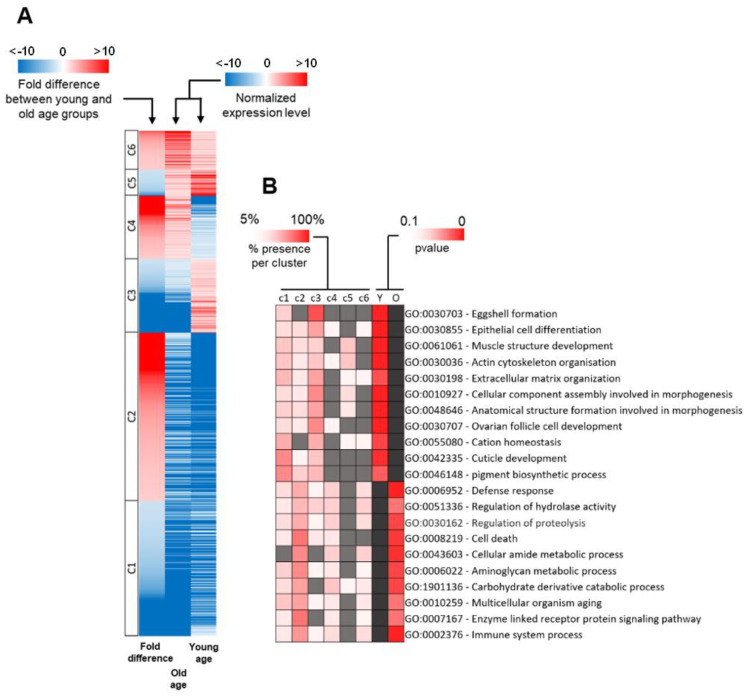
Functional classification of differentially expressed genes between young and old age groups. (**A**) Heatmap of differentially expressed genes. Expression values for the old and young age groups are shown as normalized to the mean of the expression of all genes. Blue color indicates that the expression level of a gene is lower than the mean, whereas a red color indicates a higher expression level. The fold difference column corresponds to the difference of expression of a gene between old and young age groups. The blue color corresponds to a downregulated gene in the old age group, whereas the red color corresponds to an upregulated gene in the old age group. The six gene classes are shown. (**B**) GO-term function enrichment analysis of old and young age groups and the prevalence of each GO-term in each gene class. The first six columns correspond to the six classes represented in Figure 3A (c1 to c6). The significance of the most represented GO-term in the old and young age groups is indicated by the *p*-value in the last two columns (Y for the young age group and O for the old age group). The heatmap below each gene class corresponds to the percentage of genes belonging to each GO-term that are present in this gene class. A grey tile means that less than 5% of the DEGs that belong to this GO-term are members of this gene class. A dark grey tile means that this GO-term is not overrepresented in this category.

To establish how differences in the expression of these 1184 genes determine the progression of aging, we first normalized the expression level of every DEG to the average expression level of all DEGs. We then divided all DEGs into six gene classes based on the difference of their expression level between young and old age groups (Figure 3A, Table 1). This process sorts DEGs according to their expression profile in the two age groups. The first class (c1) consisted of 328 genes that had a normalized expression lower than average in both age groups and that were upregulated in the young age group. The second class (c2) included 408 genes with lower than average normalized expression in both populations and that were upregulated in the old age group. The third class (c3) was composed of 178 genes with a lower normalized expression than average in the old age group, but higher than average in the young age group. The fourth class (c4) consisted of 154 genes that had a higher expression than average in the old age population and lower than average in the young age population. The fifth class (c5) included 63 genes that had a higher expression than average in both groups and were upregulated in the young age group. The sixth class (c6) comprised the remaining 94 genes that had a higher expression level than average in both age groups and were upregulated in the old age group.

We identified that 1040 out of 1184 DEGs (87.7%) had lower than average normalized expression in at least one of the age groups; 722 out of 1184 (60.9%) had lower than average expression in both groups. Most DEGs in c1 and c2 had low expression in both age groups. As it is mathematically easier to attain a large fold difference when the initial level of expression is low, fluctuation levels in the expression of these DEGs may not necessarily be biologically relevant to the aging process. Life-course changes in the expression levels of DEGs belonging to c3 and c4 may be more biologically relevant to aging because they present a higher expression than average in one of the age groups, but a lower expression than average in the other group. Genes in c5 and c6 are similar to those belonging to c3 and c4 in that they present a higher-than-average expression in both groups, making it mathematically harder to attain a significant fold difference between them. Sorting DEGs based on their relative expression allows us to identify DEGs that might be more biologically relevant [12] and that may be worth investigating first.

### 2.4. Functional Classification of DEGs

To determine the cellular functions that are significantly overrepresented among DEGs, we performed a gene set enrichment analysis using GSEA software (see Section 4). We selected the most significant Gene Ontology (GO)-term and showed the proportion of the different gene classes for all of them (Figure 3B). First, we focused on GO-terms that mainly include downregulated DEGs. Many genes involved in the GO-term ‘GO:0030703 Eggshell formation’ were downregulated in the old age group with 22 out of 29 DEGs (75.9%) that belonged to c3. Gene class 3 (c3) contains DEGs with lower-than-average expression in the old age group and higher-than-average expression in the young age group. Genes involved in ‘GO:0030855 Epithelial cell differentiation’ were also overrepresented among DEGs with 25 out of 52 DEGs (48.1%) belonging to c3 or c5. Genes involved in ‘GO:0061061 Muscle structure development’ and ‘GO:0030036 Actin cytoskeleton organization’ were also overrepresented among downregulated DEGs with 12 out of 27 DEGs (51.8%) and 15 out of 28 DEGs (53.6%), respectively, that belonged to c3 or c5. We also noticed the overrepresentation of genes belonging to the GO-term ‘GO:0030198 Extracellular matrix organization’ among the downregulated DEGs with 7 out of 15 DEGs (46.7%) that belonged to c3 or c5. Genes involved in two GO-terms linked to morphogenesis were also overrepresented among downregulated DEGs: ‘GO:0010927 Cellular component assembly involved in morphogenesis’ with 26 out of 36 DEGs (72.2%) that belonged to c3 or c5 and ‘GO:0048646 Anatomical structure formation involved in morphogenesis’ with 37 out of 70 DEGs (52.9%) that belonged to c3 or c5. We also observed the overrepresentation of DEGs involved in the development of ovarian follicle cells (GO term ‘GO:0030707 Ovarian follicle cell development’) with 25 out of 43 DEGs (58.1%) that belonged to c3 or c5. Many genes involved in the GO-term ‘GO:0055080 Cation homeostasis’ were downregulated in the old age group with 6 out of 11 DEGs (54.6%) belonging to c3 or c5. Interestingly, a majority of the c1 genes overrepresented among downregulated DEGs belonged to only two GO-terms: ‘GO:0042335 Cuticle development’ with 13 out of 25 DEGs (52%) and ‘GO:0046148 pigment biosynthetic process’ with 5 out of 10 DEGs (50%).

Next, we focused on GO-terms, which mainly include upregulated DEGs. Interestingly, only three GO-terms presented a majority of DEGs belonging to c4 or c6. Class 4 contains DEGs with higher-than-average expression in the old age group and lower-than-average expression in the young age group, while class 6 contains DEGS presenting higher-than-average expression in both age groups and are upregulated. Genes involved in the GO term ‘GO:0006952 Defense response’ were overrepresented among upregulated DEGs with 23 out of 58 DEGs (39.6%) that belonged to c4 or c6. The genes involved in the GO-term ‘GO:0051336 Regulation of hydrolase activity’ were also overrepresented among upregulated DEGs with 4 out of 10 DEGs (40%) belonging to c4 or c6. Genes important for the GO-term ‘GO:0030162 Regulation of proteolysis’ were overrepresented among upregulated DEGs with 5 out of 13 DEGs (38.5%), and these genes belonged to c4 or c6. A majority of c2 genes overrepresented among upregulated DEGs belonged to eight GO-terms, including ‘GO:0020037 Heme binding’ with 12 out of 27 DEGs (44.4%) and ‘GO:0008219 Cell death’ with 12 out of 20 DEGs (60%), as well as genes important for metabolic or catabolic processes, such as ‘GO:0043603 Cellular amide metabolic process’, ‘GO:0006022 Aminoglycan metabolic process’ and ‘GO:1901136 Carbohydrate derivative catabolic process’ with 10 out of 20 DEGs (50%), 13 out of 25 DEGs (52%) and 8 out of 19 DEGs (42.1%), respectively. Other c2 genes associated with ‘GO:0010259 Multicellular organism aging’, GO:0007167 Enzyme-linked receptor protein signaling pathway’, and ‘GO:0002376 Immune system process’ were also overrepresented among upregulated DEGs with 8 out of 18 DEGs (44.4%), 13 out of 21 DEGs (23.8%), and 24 out of 51 DEGs (47.1%), respectively.

Taken together, these results show that several processes are differentially regulated between the age groups. Genes involved in development or morphogenesis were mainly downregulated in the old age group, whereas genes involved in immune response, proteolysis, and cell death were mainly upregulated in the old age group. The downregulation of the genes involved in ‘GO:0030703 Eggshell formation’ and, more globally, in egg development were expected since several studies reported it [13,14,15]. The downregulation of genes involved in ‘GO:0030855 Epithelial cell differentiation’ is consistent with the expected epithelial regeneration decrease during aging [16]. The global downregulation of genes involved in ‘GO:0061061 Muscle structure development’ was also expected since it has already been reported several times [17,18]. The upregulation of genes involved in ‘GO:0006952 Defense response’ and the immune system, in general, was also expected and has been extensively studied over the last decade [14,15,19]. We also expected to find genes involved in the apoptotic process to be upregulated during aging since several studies have reported the phenomenon in *Drosophila* [13,20]. Therefore, our results are consistent with the *Drosophila* aging transcriptomic literature.

### 2.5. Overrepresentation of Genes Important for Flight Muscles and Sarcomere

Our study identified 27 genes related to the GO-term “muscle structure development” and another 28 genes related to the GO-term “actin cytoskeleton organization”. These genes were variably expressed between the young and old age groups in a whole organism context, and most of them were downregulated in old age flies. Twenty-five of them play a role in the setup and maintenance of sarcomeres (Table 2). Among these genes, we identified two transcription factors, spalt major (Salm) and eyes absent (Eya). Salm plays an important role in the fate of fibrillary flight muscle in *Drosophila* [21]. Eya is a transcription factor essential for muscle development [22]. We also identified three different actins among DEGs. Act79b is a muscle- specific actin that is mainly expressed in the tubular muscle of the thorax in adults, including the direct flight muscles [23]. Act87E is present in adults in cephalic and abdominal muscles. Act88F is the only actin expressed in indirect flight muscles [24]. We found that the heavy chain of myosin, mhc, and the two myosin light chains, mlc1 and mlc2, were downregulated in the old age group. Additionally, among DEGs, we identified 17 components of the sarcomere, including four out of the five *D. melanogaster* troponin C (TPnC25D, TPnC47D, TPnC41C, and TPnC4), present in thin muscle filament and important for the control of muscle contraction [25]. Of these four, TPnC47D had the greatest difference in the expression levels between age groups since it was downregulated by 118-fold in old age flies. This troponin C is predominantly expressed during the embryonic stage; in adults, it is expressed weakly in the abdominal muscles but not in other parts of the body [26]. TPnC4, a troponin C mainly expressed in indirect flight muscles, was also downregulated in old age flies. The only *D. melanogaster* troponin T (Up) and the only troponin I (wupA) were also downregulated in old age flies. The two tropomyosin isoforms of *D. melanogaster*, tm1 and tm2, were also downregulated in the old age group. Thus, it appears that the entire troponin/tropomyosin complex tends to have a lower expression level in the old age group vs. the young age group.

Expression of the two muscle LIM proteins (MLPs) of *D. melanogaster* (mlp60A and mlp84B) was also downregulated in old age flies. These proteins are important for the integrity of muscle structure and were present at the Z-band of the sarcomere. Particularly, mlp60A contributes to the stiffness of flight muscles [25]. We also identified that fligthin (fln) was strongly downregulated in old age flies. This protein is specifically expressed in the indirect flight muscles where it assists with the assembly of thick filament and is important for maintaining these muscles through the entire lifespan of the fly [27]. Finally, we observed that calcium/calmodulin-dependent kinase Stretchin-Mlck (strn-mlck) and obscurin unc-89, both important for setup and maintenance of indirect flight muscles [28,29], displayed weaker expression in old age flies compared to younger flies. Taken together, these results suggest that genes that maintain muscle functions and flight muscles specifically tend to be downregulated in old age flies.

### 2.6. Genes Regulating Programmed Cell Death Are Overrepresented among DEGs

Among genes differentially expressed between the young and old age groups, our study identified 20 genes important for regulating programmed cell death. Sixteen displayed higher expression levels in the old age flies compared to the young age flies. Among these 16 genes, we identified eight that trigger programmed cell death (Table 3). We identified three caspases upregulated in old age flies, including the ‘death executioner caspase related to Apopain/Yama’ (Decay), the ‘death associated molecule related to Mch2′ caspase (Damm), and the ‘Ser/Thr-rich’ caspase (Strica). Strica is an initiator of cell death, whereas Decay and Damm are effectors [30]. Decay and Damm were members of class 4, as they present a lower- than-average expression in the young age group and a higher-than-average expression in the old age group. Strica presents low expression in both old and young age groups. Decay and Damm are predominantly expressed in the digestive system of adult flies, whereas Strica is mainly expressed in salivary glands of white prepupae [31]. The upregulation of these caspases in the old age group indicates that programmed cell death is more active in old age flies as compared to younger flies. We found that the expression of the calcium channel-forming subunit Flower (Fwe) was higher in old age flies vs. young age flies. Fwe regulates aspects of eye development and can trigger programmed cell death in slow-dividing cells and in some post-mitotic neurons; it also contributes to the formation of an optimal neural network [32]. The C2H2 zinc finger transcription factor longitudinals lacking (Lola) was also expressed at a higher level in the old age group. This transcription factor plays an important role in the death of nurse cells, which is an essential part of normal embryo development. Lola is important for chromatin condensation, taking place during programmed cell death in the ovary. Old age female flies with mutated Lola accumulate nuclear material in the ovary, and the effect of this mutation becomes stronger with age [33].

Our analysis also identified upregulated DEGs that prevent apoptosis. For instance, the ‘death-associated inhibitor of apoptosis 1′ (Diap1) had high expression levels in both age groups but was further upregulated in the old age group relative to the young age (gene class 6). Diap1 inhibits the apoptotic process by preventing caspase activation [34]. The RING finger protein Ubr3 ubiquitin ligase was similarly upregulated in old age flies, but its normalized expression was lower than average in both age groups (c2). Ubr3 physically interacts with Diap1 and upregulates Diap1 activity [34]. Ubr3 and DIAP-1 act together to prevent programmed cell death during development mainly in the eye and wing imaginal discs. The nicotinamide amidase Naam was upregulated in old age flies, but its normalized expression was below average in both age groups (gene class 2). Overexpression of Naam increases the lifespan of a fly significantly [35]. Naam has also been shown to confer resistance to oxidative stress-induced cell death in neuronal populations influencing neuronal cell survival [35]. We observed that the expression of the bhlh transcription factor Hand was upregulated more than 11-fold in old age flies vs. young age. This transcription factor is important for three major cell types of the *Drosophila* circulatory system: cardioblasts, pericardial nephrocytes, and lymph gland hematopoietic cells. Hand has been shown to prevent apoptosis in cardiac cells, possibly by maintaining a differentiated state of cardiac cells; a similar function has been shown in a mouse model [36]. Finally, the Forked box transcription factor Forked head (Fwe) also had an increased level of expression through aging. Fwe is important for preventing cell death in the salivary gland, but it has also been shown to induce ectopic cell death when its function is impaired [37]. Taken together, these findings suggest that more nuanced tissue-specific changes take place in the regulation of apoptosis, rather than an overall increase in programmed cell death in all tissues, as organism age.

### 2.7. Members of Cytochrome Family Are Overrepresented among DEGs

We identified 27 genes coding for cytochrome members among DEGs (Table 4). All of them are involved in the GO-term ‘GO:0020037 Heme binding’. Eight (29.6%) are downregulated in the old age group, whereas 19 (70.4%) are upregulated. Twenty-three of them are members of the Cytochrome P450 subfamily, which consists of proteins that contain heme as a cofactor involved in oxidoreduction reactions. They are important in the clearance of several compounds and in hormone synthesis and breakdown [38]. Among DEGs related to the Cytochrome P450 subfamily, 17 out of 23 (73.9%) have at least one predicted function in the clearance of toxic chemicals. Based on their sequence, eleven have been predicted to play a role in the metabolism of insect hormones and the breakdown of toxic chemicals, although this function has not been confirmed in vivo [39,40]. These included Cyp4ac2 (gene class 1), Cyp6a21 (gene class 3), Cyp4g15 (gene class 2), Cyp4ac3 (gene class 2), Cyp28d1 (gene class 4), Cyp6d4 (gene class 2), Cyp9b2 (gene class 6), Cyp9b1 (gene class 2), Cyp9h1 (gene class 2), Cyp6a13 (gene class 4), and Cyp304a1 (gene class 2). Six members of the Cytochrome P450 subfamily have been confirmed to play a role in toxic chemicals resistance. Four are involved in Dichlorodiphenyltrichloroethane (DDT) resistance. Cyp4p1 (gene class 4) and Cyp4p3 (gene class 4) organized in a gene class on DNA [41], Cyp6w1 (gene class 4) [42], and Cyp9c1 (gene class 2) [43]. Cyp4e1 (gene class 1) has been reported to play a role in permethrin tolerance [44], and Cyp6d2 (gene class 2) plays a role in the detoxification of toxic compounds [45]. Cyp18a1 (gene class 1) does not seem to play a role in toxic chemicals resistance, but it is involved in the inactivation of hormone 20 hydroxy ecdysone and is essential in *Drosophila* metamorphosis [46]. Finally, Cyp313a1 (gene class 1), Cyp18a1 (gene class 1), Cyp309a1 (gene class 2), Cyp12a5 (gene class 2), and Cyp12e1 (gene class 2) do not have any known function [39].

Among the members of the cytochrome family that were differentially expressed during aging, we found one member of the Cytochrome b5 subfamily, namely CG6870 (gene class 5). Cytochrome b5 proteins are hemoproteins involved in electron transport [47]. However, CG6870 does not have a known function [39]. We also found a member of the Cytochrome-C oxidase subfamily, namely CG34172 (gene class 5). Cytochrome-c oxidases are oxidoreductases that catalyze the transfer of electrons from ferrocytochrome c to molecular oxygen, reducing it to water, which is accompanied by the extrusion of four protons from the intramitochondrial compartment [48]. However, CG34172 also has no known function [39]. Finally, the two last members of the cytochrome family we found, namely CG10337 (gene class 6) and CG13077 (gene class 2) with no known function [11], belonged to the Cytochrome b561 subfamily, which consists of transmembrane proteins involved in several processes, such as stress defense, iron metabolism, tumor suppression, and various neurological processes [49].

Taken together, these results show that most cytochrome members among our DEGs are upregulated during aging. Most Cytochrome P450 proteins seem to play a role in toxic chemicals resistance and are upregulated, suggesting that older flies may be more resistant than younger flies to toxic chemicals which is consistent with the upregulation of the defense response in the aged flies we observed.

### 2.8. DEGs with Unknown Function

**Figure 4:**
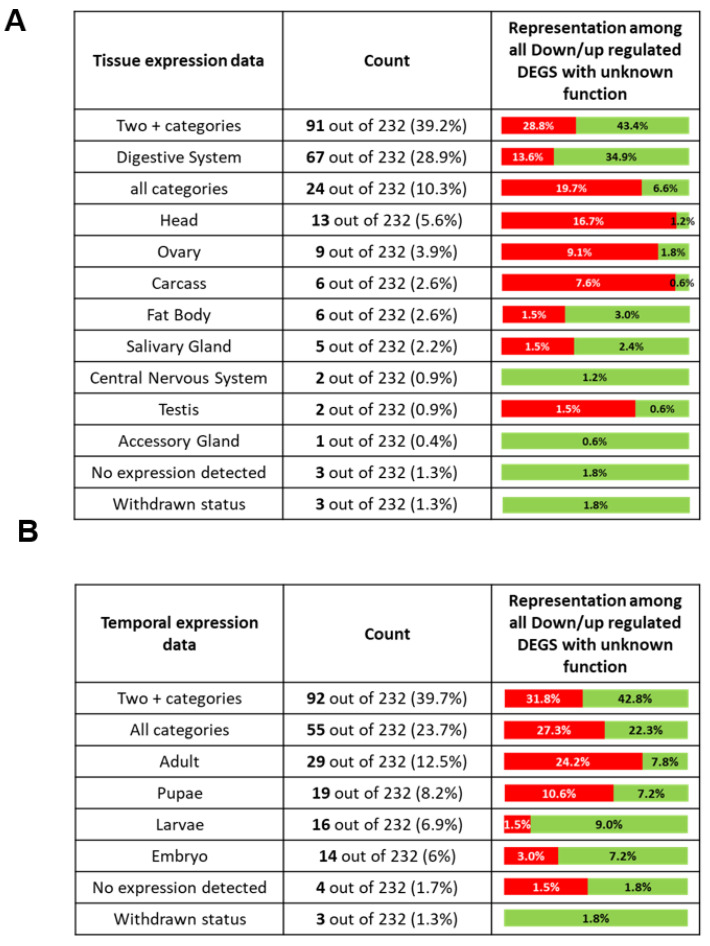
Breakdown of the RNA-seq profile of all DEGs with unknown function (modENCODE data). Two categories were addressed: “tissue expression data” (PRJNA75285) and “temporal expression data” (PRJNA75285). A gene is considered to be predominantly expressed in a specific tissue/stage if its expression level is at least two-fold higher in this tissue/stage than in the others. If the expression level of a DEG is similar in several tissues/stages, but is two-fold higher than at least one other tissue/stage, we considered this gene to be predominantly expressed in different tissues during different stages (labeled as “two + categories”). Conversely, if the fold difference of expression level of a DEG among all tested tissues/stages is lower than two, we considered the expression of this DEG to be similar in all tested tissues/stages (labeled as “all categories”). The third column of (**A**) and (**B**) corresponds to the difference of repartition between downregulated and upregulated DEGs. The red percentage corresponds to the proportion of downregulated DEGs that belong to this category, whereas a green percentage corresponds to the proportion of upregulated DEGs. Asterisks after the name correspond to the result of a two-tailed Fisher exact test. no asterisk: non-significant. (**A**) Expression of all DEGs with unknown function in different tissues. (**B**) Expression of all DEGs with unknown function during different developmental stages.

To better characterize the 232 DEGs with unknown functions, we investigated their expression profiles through RNA-seq data available on modENCODE [31,50]. We addressed two different categories: “tissue expression data” (PRJNA75285) that discriminate the expression level between different tissues and “temporal expression data” (PRJNA75285) that discriminate the expression level between different developmental stages (Figure 4). Among the 232 DEGs with unknown functions, three have a withdrawn status on Flybase, and their expression profile is not available.

We first investigated the expression profile of the 229 remaining DEGs with unknown function among different tissues (Table 5). We found that 24 out of 232 DEGs (10.2%) present a similar expression level in all tested tissues. We also determined that 91 out of 232 DEGs (39.2%) present a higher expression level in at least two different types of tissues compared to others, which means that around 50% of the DEGs with unknown function do not present a predominant expression in a specific tissue. We found that 67 out of 232 DEGs (28.9%) seem to be predominantly expressed in the digestive system compared to other tissues. Interestingly, the proportions of downregulated DEGs and upregulated DEGs predominantly expressed in the digestive system are dissimilar (*p*-value = 0.0001). Specifically, 13.6% of the downregulated DEGs are mainly expressed in the digestive system against 34.9% for the upregulated genes. Conversely, 16.7% of the downregulated DEGs are mainly expressed in the head against 1.2% of the upregulated DEGs (*p*-value = 0.0003). This suggests that the downregulated DEGs present a different expression profile from that of upregulated DEGs among different tissues.

Next, we compared the expression profile of all DEGs with unknown functions among different developmental stages (Table 5). We found that 55 out of 232 DEGs (23.7%) present a similar expression level at every stage. We also observed that 92 out of 232 DEGs (39.7%) present a higher expression level in at least two different developmental stages compared to others, which means that more than 60% of DEGs with unknown functions do not have a predominant expression during a specific developmental stage. Interestingly, we observed that 24.2% of the downregulated DEGs are predominantly expressed during the adult stage against 7.8% for upregulated DEGs (*p*-value = 0.02). Conversely, only 1.5% of the downregulated DEGs are predominantly expressed during the larval stage against 9% of the upregulated DEGs (*p*-value = 0.01). This suggests that the expression profile of the downregulated DEGs with unknown functions is different from that of the upregulated DEGs.

To better understand the roles of genes with unknown functions, we randomly selected 12 up- and downregulated genes (Table 5). First, we examined the effect of these genes on longevity by depleting the corresponding proteins using ubiquitous expression siRNAs in wild-type *Drosophila*. The knockdown of CG9084, CG31148 CG12780 and CG2505 significantly extended lifespan. Other knockdowns were either lethal during early embryonic stages (CG9090) or caused premature death (Table 5), except for the knockdown of CG11893 that did not significantly affect lifespan. The lethality observed in larval and young age flies suggests that all tested genes, but one, perform a vital function in embryonic development or in the adult organism.

Second, to determine the effect of global ectopic expression of these 12 genes, we created transgenic flies expressing recombinant proteins fused with GFP protein. To allow the transcription of a recombinant protein under the control of a GAL4 transcriptional factor, we fused cDNAs of these genes with GFP ORF and cloned them into a pUASt vector. The ubiquitous ectopic expression using the Actin-GAL4 driver 9-resultant protein in wild-type *Drosophila* of two of the 12 genes significantly extended longevity (CG13215 and CG12780), whereas 7 significantly diminished longevity or led to premature lethality (CG9090) (Table 5). Finally, the ubiquitous ectopic expression of three genes did not significantly affect longevity (CG11893, CG13311 and CG31148).

**Figure 5:**
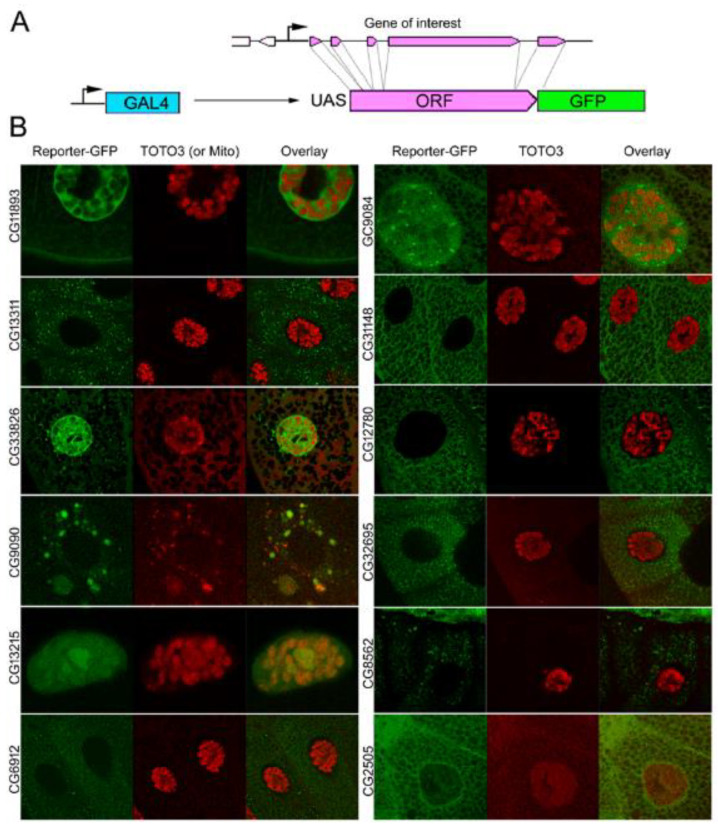
Cellular localization of twelve randomly selected genes out of the list of 232 with unknown function. (**A**) To allow transcription of the recombinant protein under the control of the GAL4 transcriptional factor, we fused cDNAs of these genes with GFP ORF and cloned them into a pUASt vector. (**B**) To study cellular localization and secretion of twelve recombinant proteins, we expressed them in larval salivary glands (SG) using a forkhead-GAL4 driver and examined them using confocal microscopy. Live, dissected larval salivary glands expressing reporter-transgenes (green) were stained with DNA-binding dye (red, shown only in Overlay) and analyzed by confocal microscopy live imaging. A single cell is shown for each experiment. We used TOTO3 staining (red) to detect nuclear DNA (chromatin) (for all except CG9090) and mitochondrial protein Tim17b (red) (for CG9090). We distinguished localization of the following proteins using morphology and co-staining: soluble nucleoplasm, chromatin, nucleolus, nucleoplasmic granules; soluble cytoplasm, secretory granules, mitochondria, perinuclear and peri-plasma membrane space and secreted when recombinant proteins accumulate in SG lumen.

Third, to study the cellular localization and secretion of the twelve recombinant proteins, we expressed them in larval salivary glands and adult midgut tissue using forkhead-GAL4 and Arm-GAL4 driver, followed by examination with confocal microscopy. We decided to start with these tissues since they are easy to stain. The localization of the resultant recombinant proteins is shown in Figure 5 and Table 5. We distinguished the location of the following proteins using morphology and co-staining: soluble nucleoplasm, chromatin, nucleolus, nucleoplasmic granules, soluble cytoplasm, secretory granules, nucleoplasmic granules, mitochondria, perinuclear and peri-plasma membrane space and secreted when recombinant proteins accumulate in sg lumen (Supplementary Figure S2). We observed that the cellular localization of each of these 12 genes was consistent by ectopic expression between larval salivary glands and adult midgut tissues (Supplementary Figure S3).

Only five of these 12 genes have a direct homolog in humans, and none have a reported subcellular localization. SLC25A3, the human homolog of CG9090, is a transmembrane protein and a member of the phosphate carrier family. PLSCR1, the homolog of CG9084, is a transmembrane protein involved in phospholipid translocation. GBA is the homolog of CG31148 and is a glucoside hydrolase. CPB1, the homolog of CG8562, is a carboxypeptidase. Finally, ACHE is the homolog of CG2505 and is an acetylcholinesterase. Taken together, these results suggest that some of these 12 genes with unknown functions may play an important role in the aging process.

## 3. Discussion

**Figure 6:**
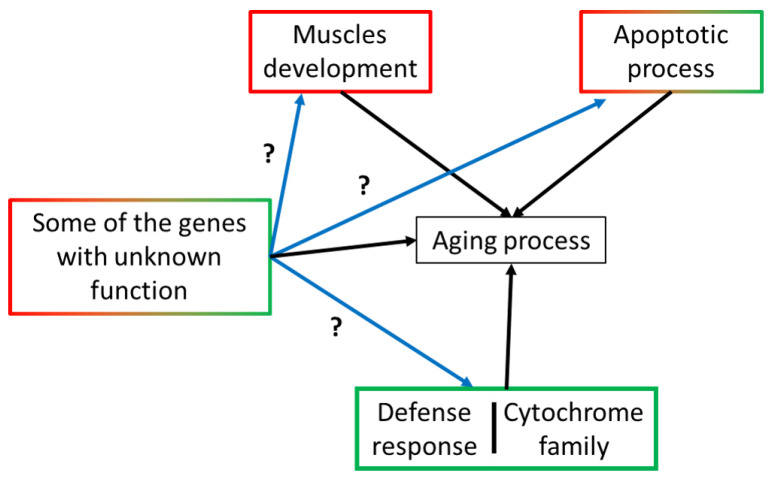
Overview of the processes that are affected during aging process. Processes contoured in red are mainly downregulated during aging while processes contoured in green are mainly upregulated during aging. Among the 12 genes tested the knockdown of five decreased longevity, whereas their expression level increased during aging. Conversely, the knockdown of one increased longevity, whereas its expression level decreased during aging. Black arrows represent known interaction while blue arrows represent potential interaction.

We identified 1184 genes differentially expressed between young age flies (three-day-old adult females) and old age flies (45-day-old females). We observed that a majority of genes overrepresented in our DEGs belong to c1 or c2 and are associated with several GO-terms. Our functional analysis revealed that only a small proportion of functions overlapped between upregulated and downregulated DEGs, suggesting the differential regulation of several processes between young and old age groups. This differential regulation likely reflects the regulatory changes responsible for the aging process (Figure 6).

Several genes involved in GO-terms and important for early-stage development were expected to be downregulated during aging, such as ‘GO:0030703 Eggshell formation’. A previous paper revealed that genes involved in egg development were significantly downregulated in flies older than 35 days compared to 2-day-old flies [13]. The downregulation of some genes involved in muscle development during aging was also expected since it has already been reported in a previous study [17]. However, we were surprised to detect a tissue-specific difference in a whole organism context. To explain, several downregulated DEGs which were identified as playing a role in muscle development/maintenance are also expressed in specific tissues. For example, we identified eight members of the troponin/tropomyosin complex among DEGs, but also six genes involved in the Z-line, A-band or M-band of the sarcomere. We also noticed the presence of five members of a muscle-specific actomyosin complex among the DEGs. Interestingly, six of these DEGs were strongly linked with flight muscles, especially indirect flight muscles.

The global upregulation of genes involved in the immune system and the defense response processes during aging has been reported in several studies [13,51,52]. However, the upregulation of several members of the cytochrome P450 family during aging is not well described in the aging literature. Here, we highlighted the presence of 23 members of the Cytochrome P450 family among our DEGs, and most of them are upregulated during aging. Interestingly, 17 of them have at least a predicted role in toxic chemical clearance, which is a part of the *Drosophila* defense response system. However, some are also downregulated during aging, which may result from a difference of expression level in different tissues that cannot be detected with the whole organism approach. We also found upregulation of several genes involved in apoptosis during aging, which is consistent with the results of several studies [13,20]. Interestingly, we found genes that trigger apoptotic processes, but also some that prevent them. This may result from a differential activation of the apoptotic processes in different tissues. We expected to see changes in the expression of genes involved in proteostasis and autophagy processes since these two processes are known to be tightly linked with aging [53,54]. However, we did not find them overrepresented among DEGs. One possibility is that since we selected 45-days old flies as an old age group to avoid “pre-death phenotype”, which is linked to the accumulation of necrotic and/or apoptotic tissues, these flies might be too young to detect differences in the expression of genes involved in proteostasis and autophagy process.

Finally, we randomly selected 12 up- and downregulated DEGs with unknown functions and knocked down their expression with siRNA to determine if they play a role in longevity (Table 5). Interestingly, we found that all but one significantly affected lifespan. Moreover, knockdown of five decreased longevity, whereas their expression level increased during aging, suggesting that they may play a positive role in the aging process. Such is the case for CG33926, which is upregulated during aging (fold difference: 33.8), and its knockdown resulted in flies living 46% less than control flies. Conversely, the knockdown of CG2505 increased fly longevity, whereas its expression level decreased during aging, suggesting that CG2505 may play a negative role in the aging process. On the other hand, CG9090 knockdown is lethal at the larval stage and is mainly expressed in young age flies (fold difference: −179.7), suggesting that it may play a role during development, rather than during the aging process. Next, we also studied the effect of the ubiquitous ectopic expression of these 12 genes on fly longevity. We found that ubiquitous ectopic expression of nine genes significantly affected longevity. However, since we did not quantify the expression level of the flies presenting ubiquitous ectopic expression, or compare it to the endogenous expression level, we cannot exclude the possibility that the increase of expression level is just too low to see an effect of overexpression for some genes. It is also possible that the GFP tag affected the function of the protein, e.g., CG9090. Both CG9090 knockdown and overexpression are lethal, which suggests that the ubiquitous ectopic expression of CG9090 affects the normal function of the endogenous protein. We then determined the subcellular localization of these 12 genes by using transgenic flies that ectopically express a GFP fused protein version of those 12 genes in salivary gland tissue or in adult midgut tissue (Supplementary Figures S2 and S3). We decided to start with these tissues since they are easy to dissect and stain. We observed that these 12 genes presented a different subcellular localization compared to others, suggesting that they play a role in different cellular compartments. However, it is possible that the GFP tag could have affected the normal localization of the protein. It is also possible that the ectopic expression of these proteins affected their localization since only 6 of 12 genes have reported expression in salivary gland tissue [31]. However, the fact that the cellular localization of a gene is similar between salivary gland tissue and adult midgut tissue shows that any effect of GFP on cellular localization is not tissue-specific. To clarify this point, further study could generate flies expressing a GFP-tagged version of these proteins expressed under the control of their own promoter and compare their expression profile to tissue-specific RNA-seq data (PRJNA75285). Finally, some of these proteins could regulate the expression of genes involved in aging process rather than having a direct role in aging (Figure 6).

## 4. Materials and Methods

### 4.1. Drosophila Strains and Genetics and Construction of Transgenic Drosophila

Genetic markers are described in [39], and stocks were obtained from the Bloomington Stock Center and Vienna Center, except as indicated (Table 5). All fly stocks were backcrossed to the y(1), w67c23(2) genetic background, which is reported to be isogenic on chromosomes I, II, and III. Each stock, prior to using it in the reported experiments, was additionally backcrossed by mating brother and sister together for 20 generations. The isogenic stock y(1), w67c23(2) was used in the aging experiments: for each replicates, 500 flies (250 virgin females and 250 males) were placed in 10 different bottles (25 males and 25 females per bottle) with each containing 10 mL of fly food and fresh yeast. Flies were cultured in a 20 °C degree incubator with a standard day/night cycle and 50% humidity. Flies were transferred to a fresh bottle every three days. To purify total RNA, 100 males and 100 females were taken among 3 days-old flies and then among 45 days-old flies. In total, 97% of the flies were still alive at day 45. While working with heat shock inducible transcription, we found that the hsp70 promoter is quite active at 25 °C, but silent at 20 °C. Thus, we assumed that 25 °C exerts a semi-stressful condition, so we decided to use 20 °C in our experiments.

To construct UAST::EGFP-transgenic reporters, we generated full-length copies of selected genes genomic fragments using PCR. Primers were designed according to the Gateway cloning system provider recommendations: N-terminal end: starts with 5′-CCAC, then start codon ATG followed by 17bp of the corresponding 5′-end sequence of the ORF: C-terminal end primer: 20 bp 3′-end sequence of ORF excluding the stop codon. We used wild-type Drosophila genomic DNA as a template for PCR. The resulting PCR products were cloned through The Drosophila Gateway™ vector cloning system (Carnegie Institution of Washington) into the corresponding vector for Drosophila transformation. The driver strain for the expression of reporter experiments was arm-GAL4 [55]. The 69B-GAL4 driver is described in [56].

### 4.2. RNA Isolation and Microarray

Total RNA was purified using the RNeasy Mini kit (Qiagen, Valencia, CA, USA) after isolation using TRIzol reagent (Life Technologies, Inc., Grand Island, NY, USA). Microarray services were provided by the UPENN Molecular Profiling Facility, including quality control tests of the total RNA samples by Agilent Bioanalyzer and Nanodrop spectrophotometry. All protocols were conducted as described in the NuGEN Ovation User Guide and the Affymetrix GeneChip Expression Analysis Technical Manual. Briefly, 100 ng of total RNA were converted to first-strand cDNA using reverse transcriptase primed by poly(T) and random oligomers that incorporated an RNA priming region. Second-strand cDNA synthesis was followed by Ribo-SPIA linear amplification of each transcript using an isothermal reaction with RNase, RNA primer and DNA polymerase (Ovation RNA Amplification System V2, NuGEN Inc., San Carlos, CA, USA). The resulting cDNA was assessed by Bioanalyzer, fragmented and biotinylated (Encore Biotin Module, NuGEN Inc., San Carlos, CA, USA), followed by the addition of 3.75 ug of labeled cDNA to Affymetrix hybridization cocktails. Target hybridization was performed on GeneChip *Drosophila* Genome 2.0 arrays (Affymetrix Inc., Santa Clara, CA, USA) according to the manufacturer’s procedures in the GeneChip^®^ Hybridization Oven 645, followed by washing and staining in the GeneChip^®^ Fluidics Station 450. Data were acquired with the GeneChip^®^ Scanner 3000 7G (Affymetrix, Inc., Santa Clara, CA, USA). Three biological replicates per group were made (young and old age). The microarray chip included 18,952 probes that targeted 13,227 different genes.

### 4.3. Microarray Data Analysis

Data analysis was performed using Partek Genomics Suite v6.5 to apply the GCRMA normalization algorithm. Differential expression analysis was conducted using Significance Analysis of Microarrays (SAM, v3.09) and the two-way ANOVA tool. SAM calculates a score for each gene based on the change in expression relative to the standard deviation of all measurements by computing a *t*-test based on the three biological replicates. SAM then performs a set of permutations to determine the false discovery rate (FDR) with an adjustment for multiple testing. The reported list of ranked genes has a ‘delta value’ which defines the threshold of false positive in the validated dataset, which was adjusted to a stringent FDR < 15% [57]. Affymetrix. cel files were imported into Partek Genomic Suite (v6.5, Partek Inc., St. Louis, MO, USA), and GCRMA normalization was applied. Data were exported to SAM (v3.09, Significance Analysis of Microarrays, Stanford University) to test for differential expression, yielding a fold change, a *p*-value based on a *t*-test realized on the three biological replicates, and an FDR. The fluorescence intensity of each probe was recorded as a log_2_ value. To determine the expression level for every gene, the formula 2^X^ was used, where X is the average fluorescence intensity of the three biological replicates. The ratio between the expression level in the young and the old age group, termed fold difference, was used to evaluate the changes of expression levels between the young and old age groups for every gene. A negative value corresponds to a gene that is downregulated in old age flies compared to young age flies, while a positive value corresponds to a gene upregulated in old age flies. A total of 1184 genes were found to be differentially expressed using cutoffs of 2-fold increase or decrease with a false discovery rate <15% and a *p*-value for the two-tailed *t*-test <0.05. Using a combination of *p*-value and FDR allowed us to identify differentially expressed genes (DEGs) while minimizing the number of false positives [57]. The fold difference establishes an arbitrary threshold for a difference of expression level between the young and old age groups that we accepted as sufficient difference to consider a gene as differentially expressed between the two groups.

### 4.4. DEG Cellular Functions and GO-Terms Overrepresentation Analysis

The main cellular functions of the 1184 genes differentially expressed through aging were determined using the PANTHER classification system [11]. This analysis sorted the 1184 DEGs depending on their known functions without any statistical analysis.

Overrepresentation of Gene Ontology-terms (GO-terms) among DEGs was determined using Gene Set Enrichment Analysis (GSEA) software, as described in Subramanian et al., and Mootha et al. [58,59]. The list of 13,227 genes included in the microarray was used. First, GSEA sorts all the genes according to their differential expression between the young and the old age groups from the most downregulated to the most upregulated in the old age group. This differential expression is based on the three biological replicates. The list created is called L. Then, GSEA calculates a score (called enrichment score (ES)) for each GO-term. The score is calculated by walking down the L list, increasing a running-sum statistic when GSEA encounters genes associated with this GO-term and decreasing it when GSEA encounters genes not associated with this GO-term. The magnitude of the increment depends on the position of the genes in the L list: the increment is higher if the gene is at the extreme of the list rather than closer to the median. The score is the maximum deviation from zero encountered in the random walk; it corresponds to a weighted Kolmogorov-Smirnov-like statistic [12]. After that, GSEA performs a set of random permutations between groups, which generates a null distribution. The *p*-value of the score of each GO-term is then calculated relative to this null distribution. Finally, GSEA calculates the FDR for each GO-term based on its score and the number of its associated DEGs. The metric used to rank genes has been set up on “Signal2Noise”. GO-terms with fewer than 4 DEGs were excluded during the analysis. Only GO-terms with *p*-value < 0.05 and an FDR < 25% were considered as overrepresented in one of our groups. To avoid redundancy, when a GO-term was included in another GO-term, only the GO-term representing the smallest subset of genes was selected. For each selected GO-term, the proportion of each gene class (c1–c6) has been calculated (Figure 3B).

### 4.5. Lifespan Experiments

To examine the how ectopic expression of each gene with unknown function affects fly longevity, we generated *Drosophila* stocks expressing individual GFP reporters under the control of strong ubiquitous GAL4 driver Actin-GAL4. Fifty newly hatched female flies and 45 newly hatched males of each genotype were placed together in bottles containing regular *Drosophila* food with dry yeast. Ten replicates were used for each experiment. All flies were switched to new bottles every two days. Deaths were counted daily. For a negative control, we used flies carrying Actin-GAL4 only. This experiment was performed at 20 °C. To study the consequences of knockdown for each gene with unknown function for *Drosophila* longevity, we established fly stocks expressing individual siRNA transgenic constructs (Table 5) under the control of strong ubiquitous GAL4 driver Actin-GAL4. As in the previous set of experiments, 50 newly hatched female flies and 45 newly hatched males of each genotype were placed together in bottles containing regular *Drosophila* food with dry yeast. Ten replicates were used for each experiment. Flies were moved to a new bottle every two days, and the number of deaths was recorded daily. For a negative control, we used flies bearing Actin-GAL4 only. This experiment was also performed at 20 °C.

## Figures and Tables

**Table 1:** Summary of differentially expressed genes (DEGs) by class. Gene class 1 and 2 correspond to DEGs with low expression compared to other genes in both young and old age groups. Gene class 3 and 4 correspond to DEGs with low expression compared to other genes in one group and a high expression in another group. Gene class 5 and 6 correspond to DEGs with high expression compared to other genes in both young and old age groups.

Gene Class	Number of Genes Included	Expression in the Young Age Group Compared to Average	Expression in the Old Age Group Compared to Average	Down- or Upregulated during Aging
1	328	Lower	Lower	Downregulated
2	408	Lower	Lower	Upregulated
3	178	Higher	Lower	Downregulated
4	154	Lower	Higher	Upregulated
5	63	Higher	Higher	Downregulated
6	94	Higher	Higher	Upregulated

**Table 2:** Sample of genes that belong to the GO-terms “Muscle structure development” and “actin cytoskeleton organization”. Fold change shown is old age flies relative to young age flies.

Gene	Differential Expression Level between ‘Old Age’ and ‘Young Age’ Flies (Fold Difference)	Gene Class	Protein Group	Human Ortholog
Sls	−29.01	1	Titin	HMCN1
MLP60A	−20.92	1	Z-line-associated protein	CSRP3
TPnC25D	−9.97	1	Troponin C	CALML6
Salm	−4.9	1	Zinc finger transcription factor	SALL1
Eya	−3.44	1	Tyrosine phosphatase	EYA2
TPnC47D	−118	3	Troponin C	CALML6
Fln	−85.68	3	A-band-located protein	/
Strn-Mlck	−75.2	3	Calcuim/calmodulin-dependent protein kinase	MYLK
Unc-89	−35.71	3	obscurin	SPEG
Act88F	−20.58	3	Actin	ACTB
TPnC41C	−19.2	3	Troponin C	CALML6
Act79B	−13.2	3	Actin	ACTB
Prm	−3.48	3	Paramyosin	/
Mlp84B	−2.79	3	Z-line-associated protein	CSRP3
Tn	−2.06	3	Z-line-associated protein	TRIM2
TPnC4	−6.56	5	Troponin C	CALM1
Tm2	−3.65	5	Tropomyosin	TPM3
Up	−3.24	5	Troponin T	TNNT2
mlc2	−3.2	5	Myosin light chain	MYL
Tm1	−3.16	5	Tropomyosin	TPM1
Fhos	−3.04	5	Formin -like	FHOD3
mlc1	−2.78	5	Myosin light chain	MYL
Act87E	−2.75	5	Actin	ACTB
mhc	−2.47	5	Myosin heavy chain	MYH
Mf	−2.44	5	A-band-located protein	/
Bt	−2.27	5	Projectin	TTN
wupA	−2.24	5	Troponin I	TNNI2

**Table 3:** Sample of genes that belong to the GO-term “Cell death”. Fold change shown is old age flies relative to young age flies.

Gene	Differential Expression Level between ‘Old Age’ and ‘Young Age’ Flies (Fold Difference)	Gene Class	Protein Group	Human Ortholog
Fkh	3.86	2	Fork head box transcription factor	FOXA2
Strica	3.84	2	Caspase	CASP3
Ubr3	2.37	2	RING finger protein	UBR3
Hand	11.25	2	bHLH transcription factor	HAND2
Lola	3.33	2	C2H2 zinc finger transcription factor	ZBTB20
Naam	3.11	2	Nicotinamide amidase	/
Fwe	3.11	2	Other calcium channel-forming subunit	CACFD1
Decay	6.72	4	Caspase	CASP3
Damm	13.09	4	Caspase	CASP3
Diap1	2.7	6	RING finger protein	BIRC2

**Table 4:** DEGs that encode for a member of the cytochrome family. In total, 23 out of 27 DEGs are related to the Cytochrome P450 subfamily. A negative differential expression level means that this gene had higher expression in young age flies compared to its expression in old age flies. A positive differential expression level means that this gene had a lower expression level in the young age flies compared to its expression in old age flies. The first part of the table includes DEGs that are downregulated during aging. The second part of the table includes DEGs that are upregulated during aging.

Gene	Differential Expression Level between ‘Old Age’ and ‘Young Age’ Flies (Fold Difference)	Gene Class	Protein Group	Function	Human Closest Ortholog
Cyp313a1	−16.6	1	Other cytochrome P450	Unknown	CYP26B1
Cyp4ac2	−7.3	1	Other cytochrome P450	Toxic chemicals breakdown (predicted)	CYP4V2
Cyp4e1	−5.0	1	Other cytochrome P450	Permethrin resistance	CYP4V2
Cyp6a21	−6.2	3	Other cytochrome P450	Toxic chemicals breakdown (predicted)	CYP3A4
Cyp305a1	−2.7	3	Other cytochrome P450	Unknown	CYP2J2
Cyp18a1	−2.2	1	Other cytochrome P450	Steroid hormone inhibition	CYP2J2
CG34172	−2.9	5	Cytochrome-C oxidase	Unknown	COX7A1
CG6870	−2.1	5	Cytochrome b5	Unknown	CYB5A
Cyp4g15	2.1	2	Other cytochrome P450	Toxic chemicals breakdown (predicted)	CYP4V2
Cyp4ac3	8.4	2	Other cytochrome P450	Toxic chemicals breakdown (predicted)	CYP4V2
Cyp4p1	7.9	4	Other cytochrome P450	DDT resistance	CYP4V2
Cyp4p3	13.2	4	Other cytochrome P450	DDT resistance	CYP4V2
Cyp6w1	2.5	4	Other cytochrome P450	DDT resistance	CYP3A4
Cyp9c1	6.1	2	Other cytochrome P450	DDT resistance	CYP3A4
Cyp6d2	31.0	2	Other cytochrome P450	Camptothecin resistance	CYP3A4
Cyp28d1	2.6	4	Other cytochrome P450	Toxic chemicals breakdown (predicted)	CYP3A4
Cyp6d4	3.2	2	Other cytochrome P450	Toxic chemicals breakdown (predicted)	CYP3A4
Cyp309a1	10.2	2	Other cytochrome P450	Unknown	CYP3A4
Cyp9b2	2.2	6	Other cytochrome P450	Toxic chemicals breakdown (predicted)	CYP3A5
Cyp9b1	2.3	2	Other cytochrome P450	Toxic chemicals breakdown (predicted)	CYP3A5
Cyp9h1	14.1	2	Other cytochrome P450	Toxic chemicals breakdown (predicted)	CYP3A5
Cyp6a13	3.7	4	Other cytochrome P450	Toxic chemicals breakdown (predicted)	CYP3A7
Cyp304a1	53.7	2	Other cytochrome P450	Toxic chemicals breakdown (predicted)	CYP2F1
Cyp12a5	2.2	2	Other cytochrome P450	Unknown	CYP24A1
Cyp12e1	4.3	2	Other cytochrome P450	Unknown	CYP24A1
CG10337	2.2	6	Cytochrome b561	Unknown	CYB561D1
CG13077	11.8	2	Cytochrome b561	Unknown	CYB561D1

**Table 5:** Cellular localization and effects of ectopic expression and knockdowns on longevity. Twelve randomly selected genes from the list of 232 with unknown function. Column 2 shows the fold change of gene expression in aged flies; 3—cellular localization; 4—secretion; 5—effects of ectopic expression on longevity compared with Actin-GAL4 *Drosophila;* 6—names of siRNA knockdown transgenes; 7—effects of knockdowns on longevity compared with Actin-GAL4 *Drosophila*. All numbers included in columns 5 (Ectopic expression effect on longevity) and 7 (Phenotype of the knockdown) have *p*-value < 0.05.

Gene	Differential Expression Level between ‘Old Age’ and ‘Young Age’ Flies (Fold Difference)	GFP-Reporter Localization	Secreted	Ectopic Expression Effect on Longevity	siRNA Transgene (Vienna)	Phenotype of the Knockdown
CG11893	4.6	Soluble nucleoplasm; Secretory granules	Yes	No	v16356 v105267	No
CG13311	44.3	Cytoplasmic particles	No	No	v101655 v51574	−17%
CG33926	33.8	Soluble nucleoplasm; Secretory granules	Yes	−11%	v107542	−46%
CG9090	−179.7	Mitochondria	No	lethal	v101848 v44297	lethal
CG13215	59.9	Nuclear chromatin and nucleolus	No	+5%	v37166 v37167	−22%
CG6912	3.7	Secretory granules	Yes	−7%	v102806 v31093	−9%
CG9084	6.0	Soluble cytoplasm; nucleoplasmic granules; Soluble nucleoplasm	No	−28%	v26908 v106414	+6%
CG31148	254.5	Soluble cytoplasm	No	No	v14698 v14697	+6%
CG12780	2.6	Secretory granules	Yes	+5%	v101969 v24613	+5%
CG32695	4.3	Cytoplasmic granules	No	−9%	v44751 v102678	−12%
CG8562	−121.6	Secretory granules	Yes	−6%	v44577 v44578	−24%
CG2505	−5.9	Perinuclear and peri-plasma membrane space	No	-−31%	v105578 v20879	+11%

## Data Availability

All data described herein are contained within this manuscript. Data are stored on external hard drives and an internal laboratory server. Raw results are freely available at https://www.ncbi.nlm.nih.gov/geo/query/acc.cgi?acc=GSE187896, accessed on 10 December 2021 or can be furnished upon request by contacting Alexei Tulin (alexei.tulin@und.edu).

## Supplementary Materials

The following are available online at https://www.mdpi.com/article/10.3390/genes12121982/s1, Figure S1: Graph representing the distribution of the 1184 Differentially expressed genes (DEGs), Figure S2: An example of recombinant protein secretion to the larval salivary gland lumen, Figure S3: Cellular localization of twelve randomly selected genes out of the list of 232 with unknown function, Table S1: Microarray raw data.
