# Supplementary material for: Age-Related Changes of Gene Expression Profiles in Drosophila
Source: Genes (Basel). 2021 Dec 14;12(12):1982. doi: 10.3390/genes12121982 (PMC8701748; doi:10.3390/genes12121982)
Supplement: Supplementary file 1 [file genes-12-01982-s001.zip › 2_Bordet_ SUPPLEMENTARY_INFORMATION_v3.pdf]

SUPPLEMENTARY INFORMATION

Age-related changes of gene expression profiles in *Drosophila*

Guillaume Bordet[2], Niraj Lodhi[1], Andrew V. Kossenkov[3], Alexei Tulin[2]

1 - Fox Chase Cancer Center, Philadelphia, PA

2 - University of North Dakota, Grand Forks, ND

3 - Wistar Institute, Philadelphia, PA

Address correspondence to:

Alexei V. Tulin, Ph.D.

Department of Biomedical Sciences

School of Medicine and Health Sciences

University of North Dakota

501 North Columbia Road, Stop 9061

Grand Forks, ND 58202

Phone: 1 701 777 4922; FAX: 1 701 777 2054

E-mail: Alexei.Tulin@und.edu

Running head: *Drosophila* expression profile during aging

Keywords: Aging processes | Microarray | Drosophila | Muscle structure | Cytochrome

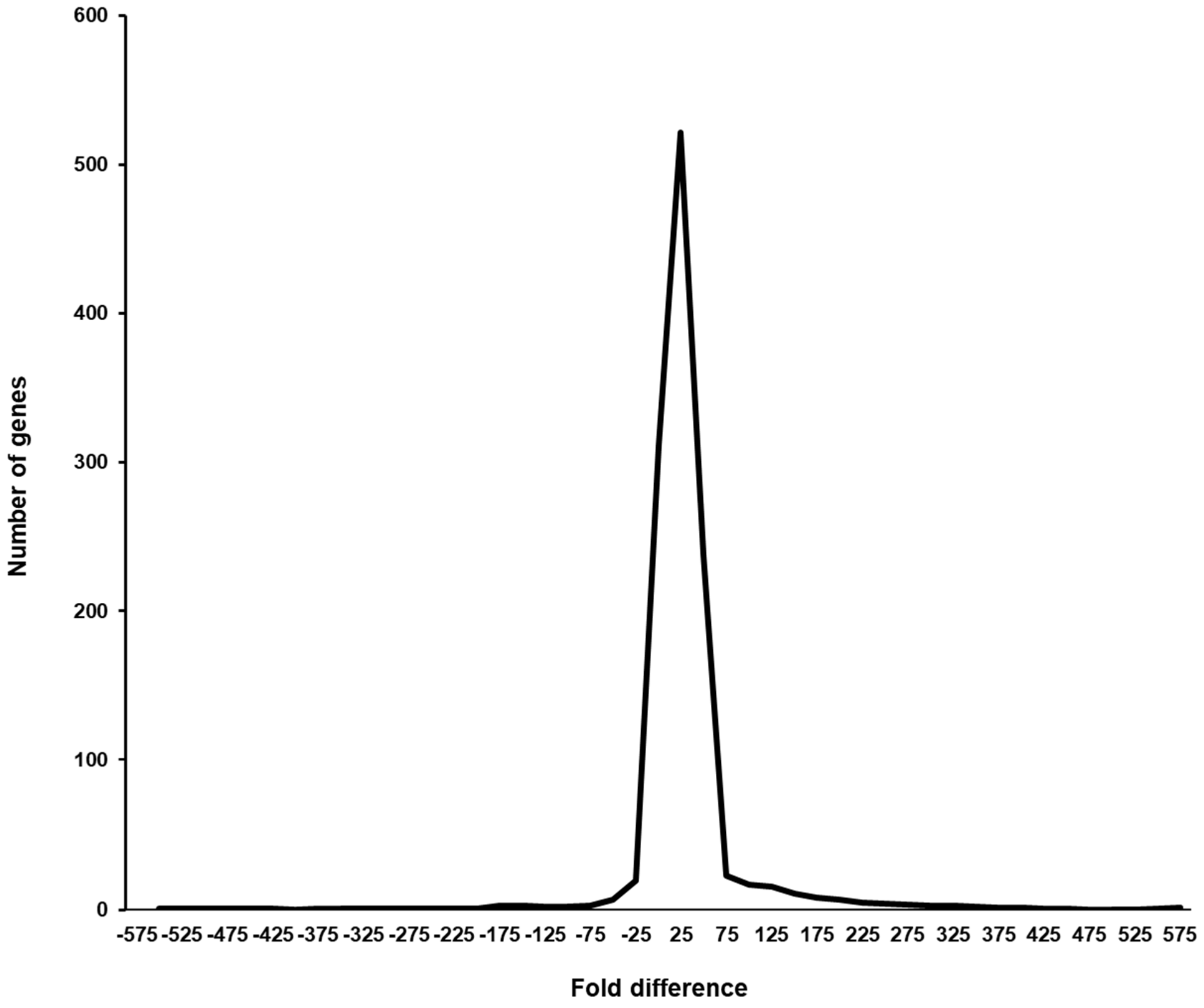

Supplemental Figure S1. Graph representing the distribution of the 1184 differentially expressed genes (DEGs), depending on the fold difference of their expression between old and young age groups. The majority of the DEGs are comprised between -25 and 75 fold.

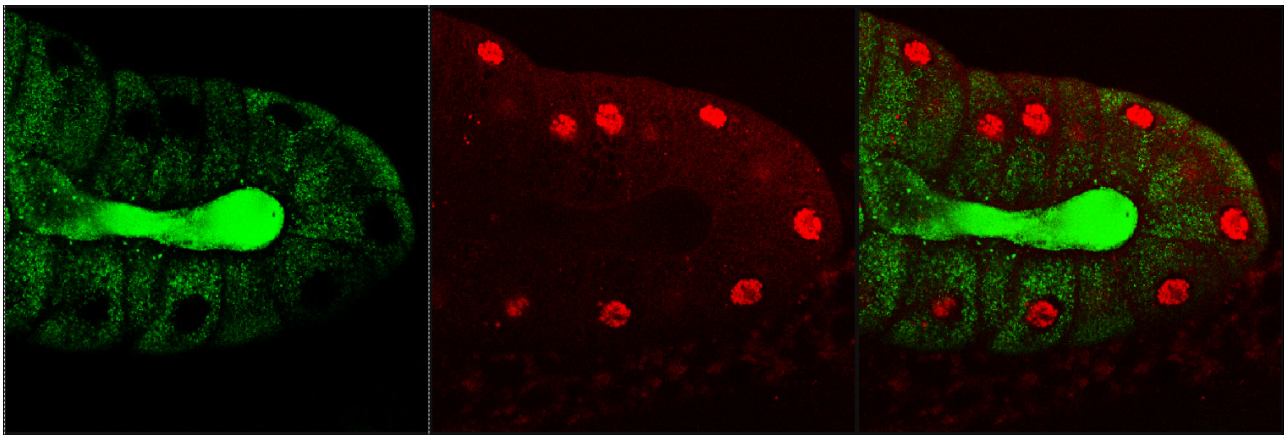

Supplemental Figure S2. An example of recombinant protein secretion to the larval salivary gland lumen.

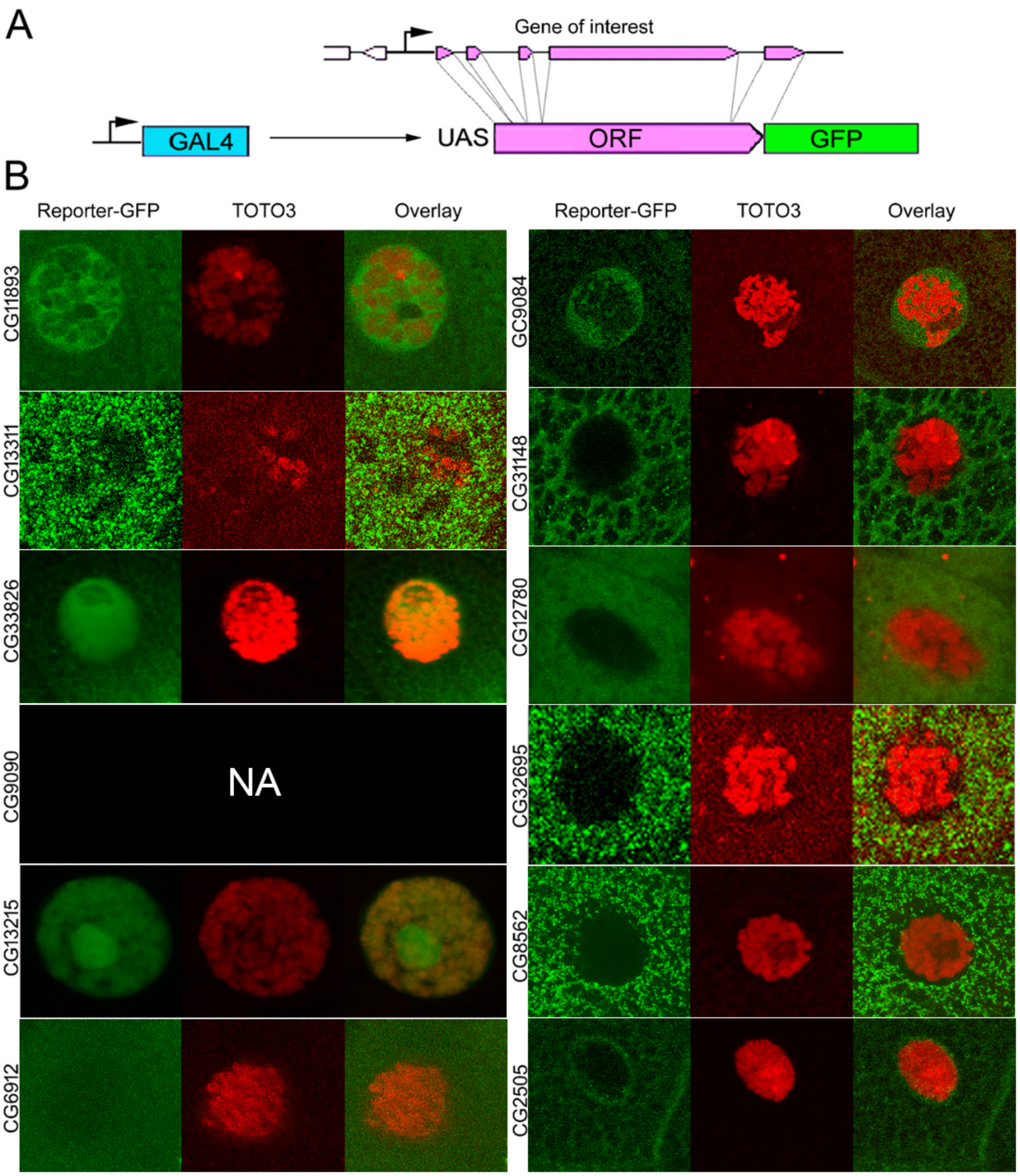

Supplemental Figure S3. Cellular localization of twelve randomly selected genes out of the list of 232 with unknown function. A. To allow transcription of the recombinant protein under control of the GAL4 transcriptional factor, we fused cDNAs of these genes with GFP ORF and cloned them into a pUASt vector. **B.** To study cellular localization and secretion of twelve

recombinant proteins, we expressed them in larval salivary glands (SG) using forkhead-GAL4 driver and examined using confocal microscopy. Live, dissected larval salivary glands expressing reporter-transgenes (green) were stained with DNA-binding dye (red, shown only in Overlay) and analyzed by confocal microscopy live imaging. A single cell is shown for each experiment. We used TOTO3 staining (red) to detect nuclear DNA (chromatin). The CG9090-GFP protein expressing animals are not surviving to adult stage. We distinguished localization of the following proteins using morphology and co-staining: soluble nucleoplasm, chromatin, nucleolus; soluble cytoplasm, secretory granules, nucleoplasmic granules, mitochondria, perinuclear and peri-plasma membrane space and secreted when recombinant proteins accumulate in SG lumen.

Supplementary Table 1

Microarray raw data. Only the differentially expressed genes (DEGs) are represented. The first column corresponds to the Entrez ID; the second column is the *Drosophila melanogaster* gene annotation symbol in Flybase; columns 3 to 5 (labeled as “Young1,” “Young2,” and “Young3”) show the measures of fluorescence intensity in the three different biological replicates of the young age group; columns 6 to 8 (labeled as “Old1,” “Old2,” and “Old3”) are the measures of fluorescence intensity in the three different biological replicates of the old age group. All measurements are presented as $\log_2$ values.
